# Intestinal Fatty Acid Binding Protein, a Biomarker of Intestinal Barrier, is Associated with Severity of Psoriasis

**DOI:** 10.3390/jcm8071021

**Published:** 2019-07-12

**Authors:** Mariusz Sikora, Albert Stec, Magdalena Chrabaszcz, Anna Waskiel-Burnat, Michal Zaremba, Malgorzata Olszewska, Lidia Rudnicka

**Affiliations:** Department of Dermatology, Medical University of Warsaw, Koszykowa 82a, 02-008 Warsaw, Poland

**Keywords:** gut barrier, intestinal fatty acid binding protein, microbiome, psoriasis

## Abstract

Alterations of intestinal microbiota play a significant role in the pathogenesis of psoriasis. Dysbiosis may cause disruption of the intestinal barrier, which contributes to immune activation by translocation of microbial antigens and metabolites. Intestinal fatty acid binding protein (I-FABP) serves as a biomarker of enterocyte damage. The aim of this study was to investigate clinical and metabolic factors affecting plasma concentration of I-FABP in patients with psoriasis. Eighty patients with psoriasis and 40 control subjects were enrolled in the study. Serum I-FABP (243.00 (108.88–787.10) vs. 114.38 (51.60–241.60) pg/ml, *p* < 0.001) and neutrophil to lymphocyte ratio (NLR; 2.59 (1.96–3.09) vs. 1.72 (1.36–47 2.11), *p* < 0.01) were significantly increased in patients with psoriasis compared to controls. A significant positive correlation was found between I-FABP and body mass index (BMI) (*r* = 0.82, *p* < 0.001), Psoriasis Area Severity Index (PASI) (*r* = 0.78, *p* < 0.001) and neutrophil to lymphocyte ratio (NLR) (*r* = 0.24, *p* < 0.001). Rising quartiles of I-FABP were associated with increasing values of BMI, PASI and NLR. The results of the logistic regression model confirmed an increased risk of higher disease severity with I-FABP concentration – odds ratio 3.34 per 100 pg/mL I-FABP increase. In conclusion, intestinal integrity in patients with psoriasis is affected by obesity, severity of the disease and systemic inflammation. The modulation of gut barrier may represent a new therapeutic approach for psoriasis.

## 1. Introduction

The gut microbiota refers to the complex community of microorganisms, covering more than 1000 different species of bacteria, viruses, fungi, and protozoa [1]. Microbiota colonizing the human gastrointestinal tract is estimated at 100 trillion microorganisms with around 1:1 microbial to human cells ratio [2].

Dysbiosis, alterations in the composition and function of the microbiota, has been implicated in the development and progression of various skin diseases such as psoriasis [3], atopic dermatitis [4], rosacea [5] and systemic sclerosis [6]. The concept of “gut–skin axis” links changes in intestinal microbiota with immune responses in the skin [7]. However, the exact mechanism of this crosstalk remains unclear. It may be attributed, at least partially, to increased gut barrier permeability [8].

The intestinal mucosa comprises a mechanical, chemical, biological, or immune barrier, and is the largest surface for interaction with microorganisms in the human body. Disruption of the intestinal barrier can cause the translocation of bacteria and their endotoxins or metabolites, which further induces or aggravates systemic inflammation [9]. Currently, gut permeability is under investigation for potential use not only in gastrointestinal diseases, but also in extraintestinal disorders such as psoriasis [10] and its comorbidities [11,12]. Intestinal barrier dysfunction is a consequence of the systemic inflammation fueled by gut dysbiosis and has been implicated as a factor contributing to the pathogenesis of psoriasis. With an increasing understanding of the relationship between the gut barrier, onset and course of psoriasis, there is an urgent need for reliable biomarkers of intestinal permeability.

The methods currently used for determination of gut barrier integrity such as histological analysis of intestinal biopsies or oligosaccharides absorption tests are complex, time-consuming and difficult to use in daily clinical practice. The intestinal barrier can also be determined indirectly by measuring blood plasma markers of gut integrity [13], such as intestinal fatty acid binding protein (I-FABP). This low molecular weight cytoplasmic protein is present exclusively in the enterocytes of the small intestine and its increased blood concentration indicates intestinal epithelial cell damage [14]. I-FABP has previously been used as a biomarker in patients with inflammatory bowel diseases [15], mesenteric ischemia [16], necrotizing enterocolitis [17], septic shock [18], acute pancreatitis [19] or acute decompensated heart failure [20]. Recently, increased I-FABP concentration has been confirmed in patients with psoriasis [10,21]. However, the association of I-FABP with the clinical course of psoriasis remains unexplored.

The aim of this study was to analyze how various clinical and metabolic factors influenced I-FABP concentration in patients with psoriasis.

## 2. Experimental Section

### 2.1. Patients

All eligible patients diagnosed with chronic plaque psoriasis admitted to our department between January 2018 and December 2018 were screened for inclusion in this study. Inclusion criteria: patients more than 18 years of age, diagnosis of stable plaque psoriasis ≥6 months, no systemic anti-psoriatic treatment in the previous 3 months). Exclusion criteria were as follows: concomitant chronic gastrointestinal disorder, history of gastrointestinal infection, dietary restrictions, intake of agents modulating gut microbiota (antibiotics, probiotics or prebiotics), unexplained weight loss and major surgery (all in the previous 3 months), chronic liver and pancreatic disease, inflammatory arthritis (rheumatoid arthritis, psoriatic arthritis, ankylosing spondylitis), congestive heart failure (NYHA class III or IV), estimated glomerular filtration rate (eGFR) of <60 mL/min/1.73 m^2^, pregnancy and breastfeeding. The control group comprised of individuals matched for age, gender and body mass index (BMI). Control group subjects followed the same exclusion criteria.

### 2.2. Intestinal Fatty Acid Binding Protein (I-FABP) Measurement

Venous blood samples were collected after an overnight 12-hour fast. Serum concentration of I-FABP was measured using a commercially available ELISA kit (EIAab, Wuhan, China) according to the manufacturer’s instruction. All measurements were performed in duplicate, and mean values were used for further analysis. Intra-assay coefficient of variation was 3.8% and inter-assay coefficient of variation was 8.6%.

### 2.3. Statistical Analysis 

All statistical analyses were carried out with STATISTICA 13.1 (StatSoft, Cracow, Poland). Data were evaluated for normality of distribution with Shapiro–Wilk test. Normally distributed variables were expressed as a mean ± standard deviation (SD) while non-normally distributed variables were expressed as a median and interquartile range (IQR). Categorical data were expressed as counts and percentages and were compared using a chi-squared test. Parametric and nonparametric continuous variables were analyzed using a Student’s t-test or Mann–Whitney U test, respectively. A correlation coefficient Spearman rank test was used to assess possible linear associations between two continuous variables. To compare the variables according to serum I-FABP quartiles, a Kruskal–Wallis test with Bonferroni corrections was used to analyze the variables that were not normally distributed and one-way ANOVA with Tukey’s post-hoc test for normally distributed variables. Logistic regression analysis was done to determine independent predictors of a severe course of psoriasis. Values of *p* < 0.05 were considered statistically significant.

### 2.4. Ethics

All participants signed an informed consent for inclusion in this study. The study was conducted in accordance with the Declaration of Helsinki, and the protocol was approved by the local medical ethical committee.

## 3. Results

Eighty patients with psoriasis and 40 control subjects were enrolled in the study. Table 1 shows the clinical and laboratory parameters of patients with psoriasis and control subjects. By study design, the two groups of participants did not differ with respect to age, sex distribution, BMI and smoking status. For laboratory parameters, an increase in neutrophil to lymphocyte ratio (NLR) was observed in patients with psoriasis (*p* < 0.01). There were no differences in lipid profile, fasting glucose, renal and liver function tests. The serum concentration of I-FABP was significantly increased in the psoriasis group compared with the control group (*p* < 0.001).

**Figure 1:**
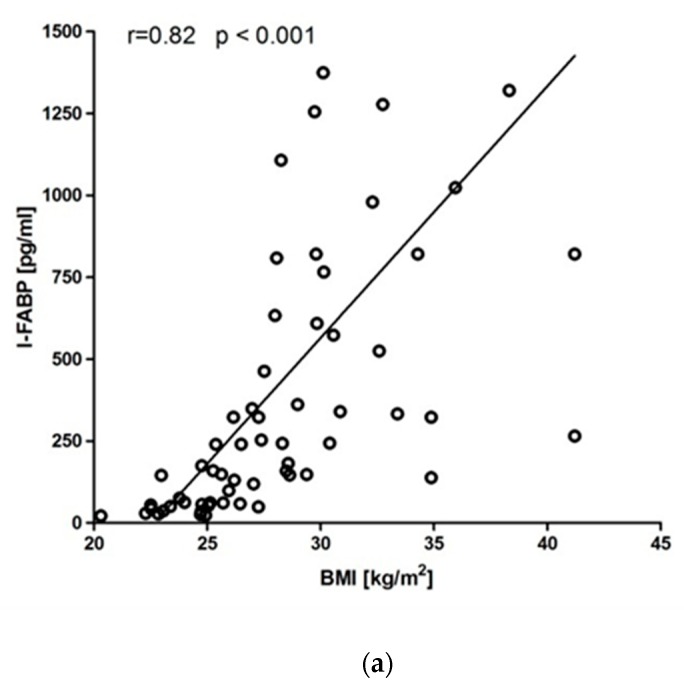

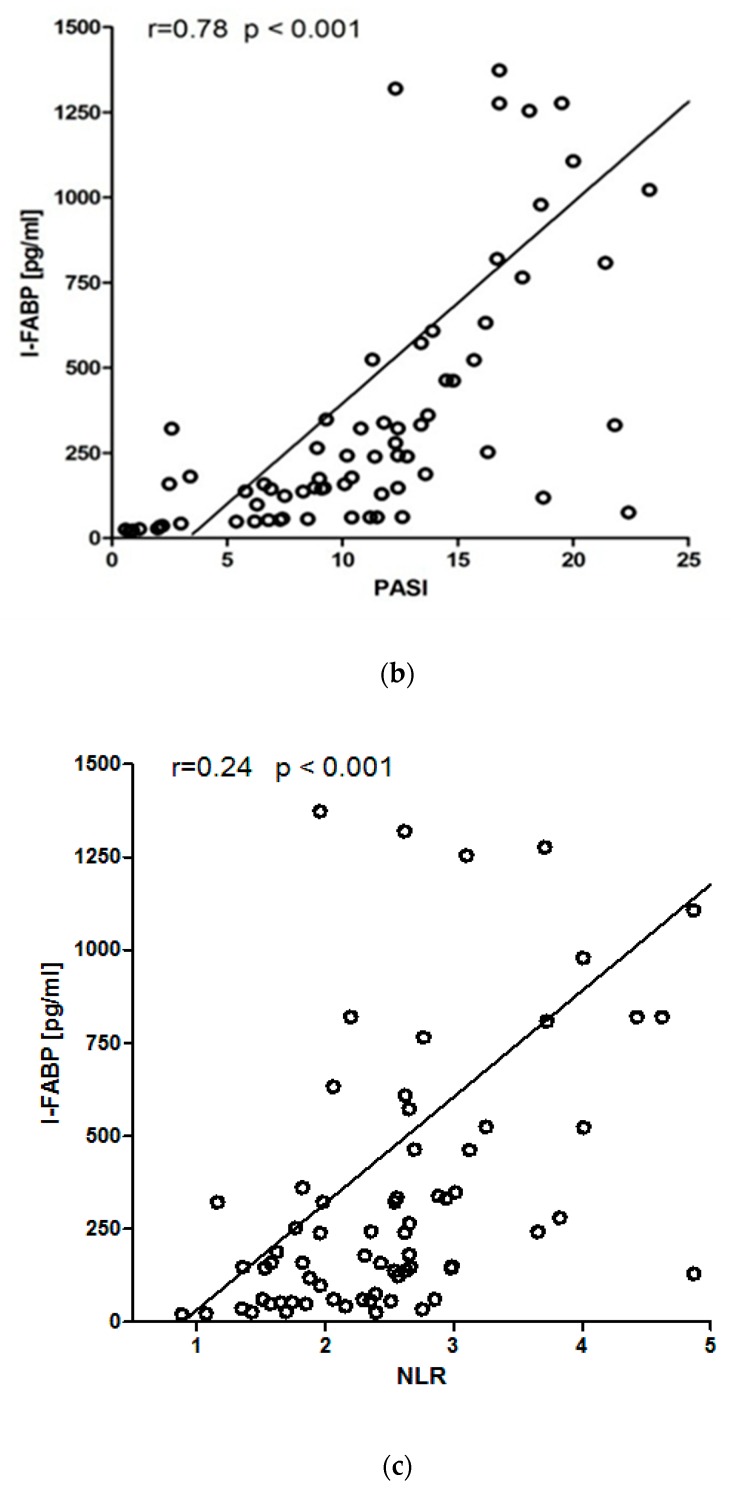
Correlations of intestinal fatty acid binding protein (I-FABP) with (**a**) body mass index (BMI), (**b**) psoriasis area severity index (PASI) and (**c**) neutrophil to lymphocyte ratio (NLR).

Table 2 summarizes Spearman’s correlation coefficients between the serum I-FABP concentration and different clinical and laboratory parameters. In patients with psoriasis, serum I-FABP significantly correlated with BMI (*r* = 0.82, *p* < 0.001, Figure 1A), PASI (*r* = 0.78, *p* < 0.001, Figure 1B) and NLR (*r* = 0.24, *p* < 0.001, Figure 1C). Since BMI showed a significant influence on I-FABP, we performed additional analyses in subgroups depending on BMI values: normal weight (18.5–24.9 kg/m^2^), overweight (25–29.9 kg/m^2^) and obese (over 30 kg/m^2^). Positive correlation between PASI and serum I-FABP was confirmed in normal weight (*r* = 0.93, *p* < 0.001), overweight (*r* = 0.89, *p* < 0.001) and obese patients (*r* = 0.57, *p* < 0.01). A similar trend of I-FABP and NLR was observed in all subgroups: normal weight (*r* = 0.58, *p* < 0.01), overweight (*r* = 0.45, *p* < 0.05) and obese patients (*r* = 0.53, *p* < 0.01). Concentration of CRP showed positive correlation with I-FABP only in psoriasis patients with a normal weight (*r* = 0.71, *p* < 0.01).

When subjects were grouped according to I-FABP quartiles (Q1 < 75.7pg/mL; Q2 75.7–242.5 pg/mL; Q3 242.6–808.5 pg/mL andQ4 > 808.5 pg/mL), rising quartiles of I-FABP concentrations (Table 3) had significantly higher values of BMI (*p* < 0.001), PASI score (*p* < 0.001) and NLR (*p* < 0.001).

In the logistic regression analysis development of moderate-to-severe psoriasis (defined by PASI ≥ 10) as the dependent variable, the concentration of I-FABP was significantly associated with disease severity. After adjustment for age, sex, BMI, smoking status, steatohepatitis, concentration of creatinine, NLR and CRP, I-FABP was an independent predictor of higher disease activity, with an odds ratio of 3.47 (95% confidence interval [CI] 1.20–10.07; *p* < 0.05) for each 100 pg/mL increase (Table 4).

## 4. Discussion

Interactions between the gastrointestinal tract and overall homeostasis involves a variety of pathways, among which intestinal barrier integrity has been intensively investigated recently. Results of our study confirmed impairment of gastrointestinal barrier in the course of psoriasis. While there is a large number of potential biomarkers of gut permeability, we decided to measure the concentration of I-FABP. This protein is the cytosolic enzyme of the enterocytes, participating in the uptake and trafficking of lipids in the intestine [14]. Blood concentration of I-FABP is very low in healthy individuals reflecting the physiological turnover rate of enterocytes, whereas it rapidly increases after intestinal epithelial damage [13]. Several experimental and clinical studies confirmed I-FABP as a surrogate marker of intestinal barrier function [15,16,17,18,19,20]. This biomarker correlates with other indicators of increased gut permeability such as lactulose/rhamnose ratio [22] and morphologic epithelial intestinal damage [23]. The reported increase in I-FABP concentration in psoriasis is in accordance with two previous studies [10,21].

The very new finding of our investigation was in determining the following factors affecting gut integrity in psoriasis: obesity (BMI), disease severity (PASI score) and systemic inflammation (NLR, CRP). Obesity, due to its rising prevalence, has become one of the leading public health concerns in the 21st century [24]. In addition to the clear connection between obesity, diabetes and hypertension, observational studies have suggested a link between increased weight and psoriasis [25]. It was shown that higher BMI contributed to the risk of psoriasis (9% increased risk of psoriasis for every 1 unit increase in BMI) [26]. Several studies have provided compelling evidence suggesting an association between obesity and an impaired gut barrier [27,28]. There is a positive correlation between waist circumference and intestinal permeability markers [29]. In a study measuring fat tissue with the use of computed tomography and dual-energy X-ray absorptiometry, gut permeability was positively correlated with the accumulation of visceral fat [29]. Interestingly, weight reduction may promote improvement of intestinal barrier function [12] as well as a decrease in the PASI [30].

Severity of psoriasis is another important independent factor influencing the integrity of the gastrointestinal barrier. We found a significant positive correlation between I-FABP concentration and PASI score in all patients and in subgroup analysis of normal weight, overweight and obese subjects. There was also a statistically significant trend showing higher values of PASI with increasing quartiles of I-FABP concentration. Additionally, a logistic regression model confirmed an increased risk of higher disease severity with an increased I-FABP concentration. The presented associations may stem from the inflammatory process underlying the pathogenesis of psoriasis. This hypothesis is supported by results of our study, showing increased NLR in patient with psoriasis and its positive correlation with I-FABP. Neutrophil to lymphocyte ratio is a simple parameter to estimate easily a patient’s inflammatory status. The prognostic value of this parameter has been proven in cardiovascular diseases [31], several types of cancers [32] and inflammatory diseases [33], such as psoriasis [34]. In our study, CRP showed a correlation with I-FABP concentration only in normal weight psoriasis patients. We did not confirm this association in individuals with higher BMI values. The data concerning the role of CRP as a marker of psoriasis severity and progression provides conflicting results [35]. While CRP is an acute-phase protein that serves as an early marker of inflammation or infection, there are significant pitfalls in the use of this biomarker in chronic inflammatory diseases. Only 17%–45.7% of patients with psoriasis present with an increased CRP concentration [36]. Additionally, obesity coexisting with the remaining components of the metabolic syndrome may increase CRP concentration independently of disease activity [35], which is probably the reason for the lack of any linear correlations between I-FABP and CRP in our study.

The pathophysiologic understanding of how intestinal barrier dysfunction contributes to the activity of psoriasis remains elusive. Dysbiosis, metabolic alterations, changes in microcirculation and inflammation promote enterocytes necrosis or apoptosis by ischemia and oxidative stress [11,37,38,39]. In turn, increased intestinal permeability allows for translocation of microorganisms, their endotoxins and metabolites via the portal vein into the systemic circulation [8]. An induced inflammatory response further intensifies damage of the barrier and “gut leakage”. This vicious circle may trigger psoriasis flare-ups.

Results of our study emphasize the interactions between the intestinal barrier, disease severity, psoriasis comorbidities and systemic inflammation. Therefore, the importance of the gut–skin axis needs to be thoroughly investigated. Interventions targeting mechanisms of gut barrier dysfunction may help prevent bacterial translocation. Maintaining the integrity of the intestinal barrier provides a new promising pathway to improve the prognosis and course of psoriasis.

## Figures and Tables

**Table 1:** Clinical and laboratory characteristics of patients with psoriasis and the control group.

	Control Group (*n* = 40)	Psoriasis (*n* = 80)	Statistical Significance
Age (years)	42.9 ± 12.7	42.5 ± 13.9	0.89
Sex, men, n (%)	28 (70%)	54 (67.5%)	0.84
BMI (kg/m^2^)	29.3 ± 6.1	29.8 ± 5.8	0.68
Smoking, n (%)	21 (52.5%)	38 (47.5%)	0.70
Steatohepatitis, n (%)	21 (52.5%)	54 (67.5%)	0.11
Psoriasis duration (years)	-	10.5 ± 6.4	-
PASI score	-	11.75 [7.45–16.8]	-
Neutrophil to lymphocyte ratio (NLR)	1.72 [1.36–2.11]	2.59 [1.96–3.09]	<0.01
Glucose (mg/dL)	87.9 ± 11.7	90.9 ± 10.5	0.78
Total cholesterol (mg/dL)	181.6 ± 18.6	188.7 ± 33.5	0.97
LDL-cholesterol (mg/dL)	106.8 ± 22.8	109.6 ± 36.2	0,76
HDL-cholesterol (mg/dL)	48.1 ± 15.8	46.6 ± 11.5	0.72
Triglycerides (mg/dL)	133.8 ± 45.7	143.8 ± 52.6	0.87
AST (U/L)	26.1 ± 13.4	27.4 ± 15.1	0.67
ALT (U/L)	32.8 ± 24.2	34.5 ± 24.8	0.75
GGT (U/L)	48.2 ± 57.3	55.9 ± 73.9	0.64
Creatinine (mg/dL)	0.84 ± 0.21	0.85 ± 0.19	0.90
eGFR, (mL/min/1.73 m^2^)	91.3 ± 25.7	89.8 ± 24.6	0.78
CRP (mg/L)	3.85 ± 4.8	4.03 ± 5.9	0.89
I-FABP (pg/mL)	114.38 [51.60–241.60]	243.00 [108.88–787.10]	<0.001

Legend: BMI—body mass index, PASI—Psoriasis Area Severity Index, AST—aspartate aminotransferase, ALT—alanine aminotransferase, GGT—gamma-glutamyltransferase, eGFR— estimated glomerular filtration rate, CRP—C-reactive protein, I-FABP—intestinal fatty acid protein; Statistically significant values are in bold.

**Table 2:** Spearman’s correlation coefficients between serum concentration of intestinal fatty acid binding protein and selected clinical and laboratory parameters in patients with psoriasis.

	All (*n* = 80)		Normal Weight (*n* = 24)		Overweight (*n* = 25)		Obese (*n* = 31)	
	*r*	*p*	*r*	*p*	*r*	*p*	*r*	*p*
Age (years)	−0.01	0.91	0.01	0.99	−0.12	0.6	0.35	0.08
BMI (kg/m^2^)	0.82	<0.001	0.12	0.62	−0.21	0.35	0.13	0.54
PASI score	0.78	<0.001	0.93	<0.001	0.89	<0.001	0.57	<0.01
NLR	0.62	<0.001	0.58	<0.01	0.45	<0.05	0.53	<0.01
Total cholesterol (mg/dL)	0.11	0.42	0.07	0.78	0.17	0.5	0.04	0.86
LDL-cholesterol (mg/dL)	0.13	0.32	0.11	0.69	0.03	0.91	0.06	0.8
HDL-cholesterol (mg/dL)	−0.27	0.35	−0.51	0.64	−0.02	0.95	−0.03	0.9
Triglycerides (mg/dL)	0.14	0.29	0.18	0.51	0.02	0.93	0.11	0.62
AST (U/L)	0.15	0.28	0.25	0.34	0.24	0.36	0.32	0.14
ALT (U/L)	0.15	0.28	0.28	0.3	0.21	0.41	0.18	0.41
GGT (U/L)	0.18	0.18	0.43	0.11	0.12	0.64	0.09	0.67
Creatinine (mg/dL)	0.28	0.14	0.04	0.87	0.18	0.49	0.37	0.09
CRP (mg/L)	0.24	0.08	0.71	0.01	0.11	0.67	0.07	0.75

Legend: BMI—body mass index, PASI—Psoriasis Area Severity Index, NLR—neutrophil to lymphocyte ratio, AST—aspartate aminotransferase, ALT—alanine aminotransferase, GGT—gamma-glutamyltransferase, eGFR—estimated glomerular filtration rate, CRP—C-reactive protein, I-FABP—intestinal fatty acid protein. Statistically significant values are in bold.

**Table 3:** Clinical and laboratory characteristics of patients with psoriasis stratified by Intestinal Fatty Acid Binding Protein concentration quartiles.

	Q1 (*n* = 20)	Q2 (*n* = 20)	Q3 (*n* = 20)	Q4 (*n* = 20)	*p*
Age (years)	38.0 [32.5–55.5]	43.0 [34.0–50.0]	40.5 [33.0–54.5]	39.5 [29.0–59.5]	0.97
Sex, men/women, n (%)	15/5 (75%/25%)	13/7 (65%/35%)	14/6 (70%/30%)	12/8 (60%/40%)	0.77
BMI (kg/m^2^)	24.35 [22.95–25.09]	26.50 [25.38–28.63]	29.99 [27.75–31.72]	32.74 [29.80–37.98]	<0.001
Smoking, n (%)	8/12 (40%/60%)	11/9 (55%/45%)	10/10 (50%/50%)	9/11 (45%/55%)	0.79
Steatohepatitis, n (%)	11/9 (55%/45%)	13/7 (65%/35%)	14/6 (70%/30%)	16/4 (80%/20%)	0.39
PASI score	5.80 [2.05–9.45]	9.05 [6.75–11.55]	13.40 [11.55–15.25]	18.70 [17.00–21.00]	<0.001
NLR	1.79 [1.47–2.37]	2.48 [1.85–2.66]	2.67 [2.45–3.07]	3.72 [2.88–4.52]	<0.001
Total cholesterol (mg/dL)	175.0 [154.0–195.5]	183.0 [157.0–200.0]	185.0 [169.0–210.0]	176.0 [162.0–205.0]	0.67
LDL-cholesterol (mg/dL)	104.4 +/− 33.0	109.4 +/− 38.3	118.2 +/− 23.0	114.2 +/− 28.60	0.71
HDL-cholesterol (mg/dL)	46.5 [43.0–51.0]	40.0 [39.0–42.0]	45.0 [40.5–48.5]	42.0 [38.0–45.0]	0.07
Triglycerides (mg/dL)	120.0 [100.5–148.0]	122.0 [87.0–170.0]	145.0 [101.0–190.0]	133.0 [106.0–165.0]	0.71
AST (U/L)	23.5 [20.0–29.5]	23.0 [21.0–26.0]	21.0 [17.5–29.0]	22.0 [17.0–24.0]	0.66
ALT (U/L)	26.5 [24.5–33.5]	25.0 [19.0–50.0]	29.0 [15.5–40.5]	25.0 [19.0–30.0]	0.53
GGT (U/L)	30.0 [24.0–46.0]	30.0 [24.0–44.0]	30.5 [16.5–56.0]	22.0 [16.0–36.0]	0.5
Creatinine (mg/dL)	0.76 +/− 0.15	0.82 +/− 0.13	0.95 +/− 0.23	0.88 +/− 0.23	0.25
CRP (mg/L)	1.92 [0.59–4.30]	2.40 [0.74–5.40]	0.92 +/− 3.1 [1.04–5.07]	3.62 [1.06–6.35]	0.45

I-FABP—intestinal fatty acid protein, Q—quartile, BMI—body mass index, PASI—Psoriasis Area Severity Index, NLR—neutrophil to lymphocyte ratio, AST—aspartate aminotransferase, ALT—alanine aminotransferase, GGT—gamma-glutamyltransferase, CRP—C-reactive protein. Statistically significant values are in bold.

**Table 4:** Logistic regression analysis showing an odds ratio for the risk of moderate-to-severe disease in patients with psoriasis.

Model	I-FABP (per 100 pg/mL Increase)	OR	95% CI	*p* Value
Model 1	Adjusting for age and sex	3.34	1.68–6.65	<0.001
Model 2	Adjusting for age, sex, BMI, smoking and steatohepatitis	3.53	1.56–8.03	<0.01
Model 3	Adjusting for age, sex, BMI, smoking, steatohepatitis, creatinine, NLR and CRP	3.47	1.20–10.07	<0.05

I-FABP—intestinal fatty acid protein, OR—odds ratio, CI—Confidence Interval, BMI—body mass index, CRP—C-reactive protein, NLR—neutrophil to lymphocyte ratio.
